# High rate of inappropriate blood transfusions in the management of children with severe anemia in Ugandan hospitals

**DOI:** 10.1186/s12913-018-3382-5

**Published:** 2018-07-18

**Authors:** Robert O. Opoka, Andrew S. Ssemata, William Oyang, Harriet Nambuya, Chandy C. John, James K. Tumwine, Charles Karamagi

**Affiliations:** 1 Department of Paediatrics and Child Health, College of Health Sciences, Makerere University, P. O. Box, 7072 Kampala, Uganda; 2 Department of Psychiatry, College of Health Sciences, Makerere University, Kampala, Uganda; 3 Children’s ward, Lira Regional Referral Hospital, Lira, Uganda; 4 Nalufenya Children’s ward, Jinja Regional Referral Hospital, Jinja, Uganda; 5 Ryan White Center for Pediatric Infectious Disease and Global Health, Indiana University School of Medicine, Indiana, USA

**Keywords:** Appropriate use, Blood transfusion, Severe anemia

## Abstract

**Background:**

Severe anaemia (SA) is a common reason for hospitalisation of children in sub-Saharan Africa but the extent to which blood transfusion is used appropriately in the management of severe anemia has hitherto remained unknown. We report on the use of blood transfusion in the management of anemic children in two hospitals in Uganda.

**Methods:**

Inpatient records of children 0–5 years of age admitted to Lira and Jinja regional referral hospitals in Uganda were reviewed for children admitted on selected days between June 2016 and May 2017. Data was extracted on the results, if any, of pre-transfusion hemoglobin (Hb) level, whether or not a blood transfusion was given and inpatient outcome for all children with a diagnosis of ‘severe anemia’. Qualitative data was also collected from health workers to explain the reasons for the clinical practices at the two hospitals.

**Results:**

Overall, 574/2275 (25.2%) of the children admitted in the two hospitals were assigned a diagnosis of SA. However 551 (95.9%) of children assigned a diagnosis of SA received a blood transfusion, accounting for 551/560 (98.4%) of the blood transfusions in the pediatric wards. Of the blood transfusions in SA children, only 245 (44.5%) was given appropriately per criteria (Pre-transfusion Hb *≤* 6 g/dL), while 306 (55.5%) was given inappropriately; (pre-transfusion Hb not done, *n* = 216, or when a transfusion is not indicated [Hb > 6.0 g/dl], *n* = 90). SA children transfused appropriately per Hb criteria had lower inpatient mortality compared to those transfused inappropriately, (7 (2.9%) vs. 22 (7.2%), [OR 0.4, 95% CI 0.16, 0.90]). Major issues identified by health workers as affecting use of blood transfusion included late presentation of SA children to hospital and unreliable availability of equipment for measurement of Hb.

**Conclusion:**

More than half the blood transfusions given in the management of anemic children admitted to Lira and Jinja hospitals was given inappropriately either without pre-transfusion Hb testing or when not indicated. Verification of Hb level by laboratory testing and training of health workers to adhere to transfusion guidelines could result in a substantial decrease in inappropriate blood transfusion in Ugandan hospitals.

## Background

Severe anaemia (SA), is a common public health problem in resource limited settings, especially in children under 5 years of age. It accounts for 9.7–29% of total paediatric admissions and 8–17% of hospital deaths in sub-Saharan Africa [1–6]. SA is a clinical condition commonly caused by severe malaria, acute bacterial infections, micronutrient deficiencies, malnutrition and sickle cell anaemia working singly or in combination [3, 7, 8]. Regardless of the cause, appropriate management involves laboratory confirmation of severity of hemoglobin (Hb) level and prompt correction of the severe anemia by blood transfusion [9]. Access to safe and reliable supply of blood is a major challenge in resource limited settings. Therefore, in order to preserve this scarce resource and reduce the risk of transfusion transmitted infections, a conservative approach is adopted in the guidelines recommended by World Health Organisation [9]. In most resource limited settings a threshold Hb of *≤* 6.0 g/dL is used for transfusion [10].

However, adherence to the guidelines is variable [11]. Blood transfusions are often given without laboratory confirmation of Hb level [12] or documented indications [13], and patients with indications for transfusions are not given blood [14]. Most of the studies that examined clinical use of blood amongst children have done so in children with a known pre-transfusion Hb level [4, 6, 15] usually under controlled research settings [11]. However in routine clinical practice the extent of implementation of the transfusion guidelines in the management of children with suspected SA is not well characterised. In addition, the factors and context specific issues associated with implementation of transfusion guidelines were also not well known. We describe the extent of appropriateness of use of blood transfusion in children admitted with a diagnosis of severe anemia in two regional referral hospitals in northern and eastern Uganda.

## Methods

### Design

The study was a retrospective review of in-patient hospital records over a 12-month period. A qualitative study was also conducted to provide context and explanation to the quantitative findings.

### Study site

The study was performed at the children’s wards of Jinja and Lira Regional Referral Hospitals located in the eastern and northern regions of Uganda. Both are public, free-for-care hospitals with the capacity to manage children with SA. Jinja hospital serves an area of seasonal malaria transmission intensity around the Lake Victoria region while Lira hospital serves an area of all year high malaria transmission around the Lake Kyoga region [16]. The hospital laboratories perform malaria tests (blood smears and rapid diagnostic tests), complete blood counts, Hb measurement by Hemacue (Jinja only), stool and urine microscopy and human immunodeficiency virus (HIV) antibody testing. Blood transfusion services are available in both hospitals.

In Uganda, blood used in public and private funded health facilities is provided by the Uganda Blood Transfusion Service. Blood is obtained from voluntary, anonymous donors and sent to one of the seven regional blood transfusion centres for testing and preparation before distribution to health units for use. Lira hospital is serviced by the Lira Regional Centre and Jinja hospital is serviced by the Jinja Regional Centre. Both regional centres are located in the respective hospitals.

### Data collection

In both hospitals, children are first seen in an outpatient section where patients that require admission are identified and taken to the paediatric wards. Once on the ward, the patients are reviewed by clinicians and managed accordingly. All management decisions and procedures are documented in the patient’s file. The files are kept by the ward nurse and taken to the records department for storage and future retrieval at the end of hospitalization. The hospital presently uses International Statistical Classification of Diseases and Related Health Problems 10th Revision (ICD 10) to classify diseases.

### Study procedures

Between June 2016 and May 2017, de-identified paediatric inpatient records for each hospital were reviewed every 4th day beginning at a randomly selected day within the first 5 days of the month. On each selected day we included all admissions to the paediatric wards between 8.00 am and 8.00 am of the following day. Records of children aged 0–5 years were followed up and reviewed at the end of hospitalization. The records reviewed included inpatient files, referral notes from other health units, laboratory request forms and results of investigations. A data extraction form was used to extract the de-identified data on demographics, final diagnosis, clinical care and inpatient outcome for the children. For all children with a diagnosis of ‘severe anemia’, we recorded results, if any, of pre-transfusion Hb level and whether or not the child was given a blood transfusion. Patients who had severe anemia due to surgical conditions or known chronic conditions such as heart disease, tuberculosis and cancer were excluded.

### Diagnosis of severe anemia and indication for blood transfusion

According to the Uganda Clinical Guidelines [17], blood transfusion is indicated if Hb < 4 g/dl or if Hb is 4–6 g/dl with signs of cardiac failure. Since it was difficult to ascertain from the records the precise indication of each transfusion, we considered any transfusion for Hb ≤ 6 g/dl as appropriate.

### Analysis

All data were entered in file maker and analysis was done using STATA version 14.1 (StataCorp, USA) statistical software. The prevalence of severe anemia was estimated from the proportion of children admitted with SA. Clinical characteristics were compared using Pearson Chi-square for means and Wilcoxon rank-sum tests for median. Appropriateness of blood transfusion was assessed using the Uganda Clinical Guidelines (2012) [17]. Inpatient outcomes (deaths) were compared to accuracy of SA diagnosis and adherence to transfusion guidelines.

#### Qualitative study

In order to provide context for the findings of the quantitative study, we carried out Focus Group Discussions (FGDs) and Key Informant Interviews (KIIs). FGDs were organized for nurses and clinicians in the children’s unit, and the technicians in laboratory and the blood bank. KIIs were done for the hospital pharmacist, clinician and nurse in-charges of the outpatients and children’s wards, and the hospital administrator. The FGDs and KIIs were conducted using a semi-structured guide by one of the authors (ASS), who is well versed with qualitative methods. The questions focused on three broad themes: 1) what contributes to the burden of SA? 2) what factors affect use of blood transfusion in children with SA? and 3) what needs to be done to improve the care of children with SA? The data was coded and sub-themes and categories were developed to explain and contextualize the management care practices.

### Ethics, consent and permissions

Ethical approval was granted by the School of Medicine and Research Ethics Committee, Makerere University (Reference Number: 2015–097) and the Uganda National Council of Science and Technology (HS 2055). Administrative permissions to access and review hospital records was granted by the office of the Directors of Jinja and Lira hospitals. Informed written consent was obtained for participants in the FGDs and KIIs.

## Results

**Fig. 1:**
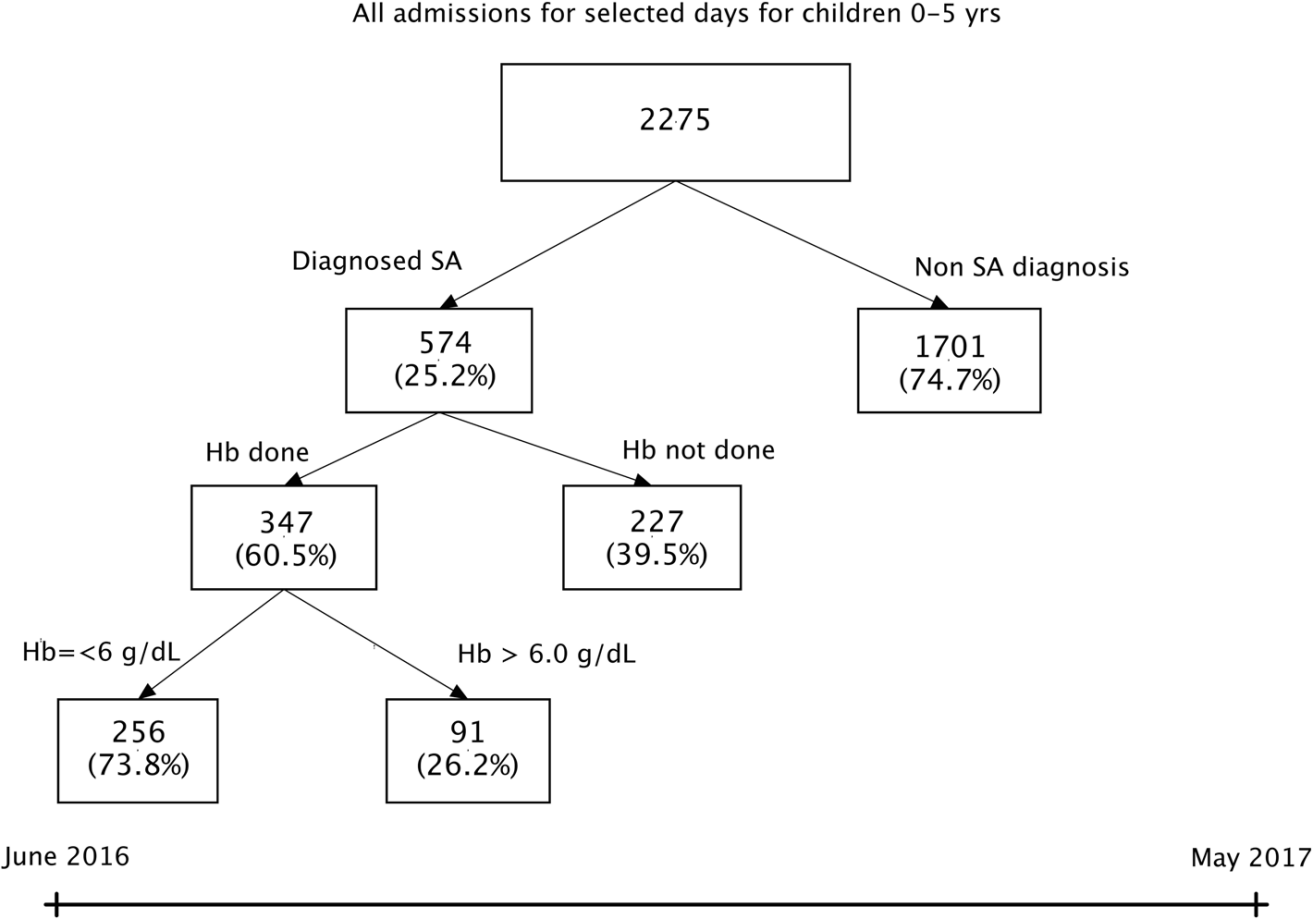
Study profile of children 0–5 years of age admitted in Lira and Jinja Hospitals on selected days between June 2016 and May 2017

A total of 2275 children aged 0–5 years were admitted to Lira and Jinja hospitals during the study period. The median age was 1.5 years (IQR 0.75, 3.0). Fever was the most common presentation while malaria test (blood smear or rapid diagnostic test) was the most common investigation done (Table 1). Overall, 574/2275 (25.2%) were assigned a diagnosis of SA. Hb testing was done for 347 (60.5%) of the children with SA diagnosis, of which only 256 (73.8%) had a pre-transfusion hemoglobin (Hb) level *(≤* 6 g/dL) (Fig. 1). The prevalence of assigned diagnosis of SA was higher in Lira than Jinja Hospital (33.5% vs. 20.5% respectively) (Table 1). The overall all-cause mortality in the two hospitals was 4.0% (Table 1).

**Table 1:** Clinical characteristics, management and outcome of children 0–5 years of age admitted in Lira and Jinja Hospitals on selected days between June 2016 and May 2017

	Lira hospital *N* = 819	Jinja hospital *N* = 1456	Overall *N* = 2275
Age, median (IQR)	1.3 (0.66, 2.6)	1.6 (0.75, 3.0)	1.5 (0.75, 3.0)
Sex, n (% male)	450 (54.9%)	815 (56.0%)	1265 (55.6%)
Presentation, n (%)			
Fever	784 (95.7%)	1271 (87.3%)	2055 (90.3%)
Cough	515 (62.9%)	879 (60.4%)	1394 (61.3%)
Vomiting	258 (31.5%)	510 (35.0%)	768 (33.8%)
Convulsions	106 (12.9%)	168 (11.5%)	274 (12.0%)
Duration of illness days, mean (SD)	2.7 (1.5)	3.3 (2.6)	3.1 (2.2)
Temperature, mean (SD)	37.9 (0.9)	37.7 (1.3)	37.8 (1.2)
Laboratory tests done, n (%)			
Hb^a^ measurement	287 (64.6%)	474 (32.6%)	761 (33.5%)
Malaria test (RDT^b^or microscopy)	600 (73.3%)	1129 (77.5%)	1729 (76.0%)
Complete Blood Count	238 (29.1%)	357 (24.5%)	595 (26.2%)
Others^c^	11 (1.3%)	02 (0.2%)	13 (0.6%)
Treatments given, n (%)			
Blood transfusion	266 (32.5%)	294 (20.2%)	560 (24.6%)
Antimalarials	590 (72.0%)	845 (58.0%)	1435 (63.1%)
Antibiotics	797 (97.3%)	1148 (78.8%)	1945 (85.5%)
Severe anemia^d^	274 (33.5%)	300 (20.6%)	574 (25.3%)
Duration of hospitalization, median (IQR)	3.0 (2.0, 4.0)	3.00 (2.0, 5.0)	3.0 (2.0, 5.0)
Outcome			
Died	30 (3.7%)	60 (4.1%)	90 (4.0%)

^a^*Hb* Haemoglobin

^b^*RDT* Rapid Diagnostic Test

^c^These included: Urinalysis, stool analysis, sickling test, HIV serology, Blood sugar

^d^Diagnosis of severe anaemia made by the clinician on basis of patient having severe pallor

### Clinical characteristics of children managed as cases of severe anemia

The median age of children with an assigned diagnosis of SA was higher than for those without SA, (2.0 vs. 1.4 years *P* < 0.001) (Table 2). SA cases tended to present more at night than non-SA cases, 29.9% vs. 18.4% respectively (Table 2). Severe malaria (*n* = 524, 91.3%) and presumed septicaemia (*n* = 207, 36.1%) were the most common clinical diagnoses associated with SA (Table 2). The majority of both SA and non-SA related deaths occurred at night, 62.5% vs. 55.3% respectively (Table 2).

**Table 2:** Clinical characteristics and inpatient outcomes of severe anemia (SA) vs. None SA patients at Lira and Jinja Hospitals

	SA *N* = 574	Non SA *N* = 1701
Age, median (IQR^a^)	2.0 (1.1, 3.5)	1.4 (0.6, 2.5)
Sex, n (% male)	335 (58.4%)	930 (54.7%)
Presentation		
History of Fever	550 (95.8%)	1505 (88.5%)
Cough	359 (62.5%)	1035 (60.8%)
Time of admission	(*N* = 260)	(*N* = 903)
Day (8.01.am to 4.00 pm)	134 (51.5%)	567 (62.8%)
Evening (4.01 pm to 9.00 pm)	49 (18.8%)	170 (18.8%)
Night (9.01 pm to 8.00 am)	77 (29.6%)	166 (18.4%)
Clinical Diagnosis^b^		
Severe Malaria	524 (91.3%)	911 (53.6%)
Septicaemia	207 (36.1%)	696 (40.9%)
Severe pneumonia	87 (15.2%)	516 (30.4%)
Severe Acute Malnutrition	12 (2.1%)	85 (5.0%)
Sickle cell Disease	78 (13.6%)	77 (4.5%)
Transfused	551 (95.9%)	9 (0.5%)
Laboratory tests done, n (%)		
Hb measurement	347 (60.5%)	414 (24.3%)
Malaria test (RDT or microscopy)	466 (81.2%)	1263 (74.3%)
Complete Blood Count	228 (39.8%)	367 (21.6%)
Outcome		
Discharged	483 (84.2%)	1419 (83.4%)
Transferred to another hospital	22 (3.8%)	12 (0.7%)
Left prior to planned discharge	37 (6.4%)	212 (12.5%)
Died	32 (5.6%)	58 (3.4%)
Time of death	N = 24	*N* = 38
Day (8.01.am to 4.00 pm)	7 (29.2%)	12 (31.6%)
Evening (4.01 pm to 9.00 pm)	2 (8.3%)	5 (13.1%)
Night (9.01 pm to 8.00 am)	15 (62.5%)	21 (55.3%)
Duration of hospitalization, median (IQR)	3.0 (2.0, 5.0)	3.0 (2.0, 5.0)

^a^*IQR* Inter Quartile Range

^b^Diagnoses as listed on the charts the by the clinician and not necessarily confirmed by laboratory testing

### Blood transfusion and inpatient outcome

Overall, 551/574 (95.9%) of children diagnosed as SA received a blood transfusion, accounting for 98.4% of all transfusions in the children 0–5 years in the two hospitals (Table 3). Of the blood transfusions given to children assigned a diagnosis of SA (44.5%) were given appropriately as per guidelines (pre-transfusion Hb *≤* 6 g/dL), while 306 (55.5%) were given inappropriately, (pre-transfusion Hb not done, *n* = 216, or transfusion not indicated [Hb > 6.0 g/dl], *n* = 90) (Table 3). The mean age, sex distribution and assigned comorbid diagnoses were similar between children transfused appropriately Hb criteria and children transfused inappropriately (Table 3). Of the SA children not transfused, 22 were referred to other centres because of shortage of blood while one child died before receiving a blood transfusion. Blood transfusion practices were similar in Jinja and Lira hospitals.

**Table 3:** Clinical characteristics and inpatient outcome of severe anemia patients according to adherence to Hb threshold for transfusion at Lira and Jinja Hospitals

	Transfused per Hb criteria (Hb *≤* 6.0 g/dl) *N* = 245	Not transfused per Hb criteria (Hb > 6.0 g/dl) *N* = 90	Transfused presumptively (Hb not done) *N* = 216
Age, median (IQR^a^)	2.5 (1.3, 3.7)	2.0 (1.0, 3.0)	2.0 (1.1, 3.5)
Sex	145 (59.2%)	54 (60.0%)	120 (55.7%)
Median Hb (IQR)	4.2 (3.4, 5.0)	7.5 (6.5, 8.4)	–
Co-morbid diagnoses^b^			
Malaria	230 (93.9%)	74 (82.2%)	204 (94.4%)
Malaria positive	165 (67.3%)	49 (54.4%)	124 (57.4%)
Sepsis	106 (43.3%)	39 (43.3%)	53 (24.5%)
Pneumonia	35 (14.3%)	16 (17.8%)	31 (14.2%)
Outcome			
Died	7 (2.9%)	7 (7.8%)	15 (6.9%)

^a^*IQR* Inter Quartile Range

^b^Diagnoses as listed on the charts the by the clinician and not necessarily confirmed by laboratory testing

SA children transfused appropriately per Hb criteria had lower odds of inpatient deaths compared to children transfused inappropriately, (7/245 (2.8%) vs. 22/306 (7.1%), [OR 0.4, 95% CI 0.16, 0.90]), *P* = 0.03).

### Qualitative study

Two FGDs and 4 KIIs were conducted in each of the two hospitals from which the following key themes and quotes emerged.

#### Burden of SA and its impact on the health system

SA was perceived to be a common problem affecting children. The high burden of SA was attributed to the high burden of malaria and sickle cell disease in these areas compounded by delays in seeking care.

Quote:

‘*The reason (for the high burden of SA) is because this region is heavily infested with malaria*.’ Clinician working in the outpatients department

‘….*so they delay in the lower health units and are only referred here late when the anemia has become severe.*’ Head Nurse of the Hospital

#### Factors affecting use of blood transfusion in SA children

The major obstacles in use of blood transfusion in the management of SA children included lack of reliable measurement of Hb, staffing shortages, late presentation of SA patients to hospital and frequent stock outs of blood. Hb measurement was unreliable because the Coulter Counter machines frequently broke down or lacked reagents, while point-of-care equipment for measuring Hb like hemocue are not always available. Staffing shortages were more acute during evening and night shifts when 48.5% of the children with SA patients presented to the hospitals.

Quotes:

‘*our Coulter Counter machine is not constant .. it is overworked, because of the over whelming patients, so it breaks down frequently.*’ Hospital administrator

*‘Hemocue is a simpler way (of measuring Hb) but government cannot buy because the cuvettes are extremely expensive.’* Laboratory technician

‘… *Many SA patients come late in critical condition which makes it difficult to carry out investigations as necessary. So we transfuse immediately without delay.*’ Clinician working on children’s ward

*‘…blood stock outs are very frequent because of the high transfusion rates.*’ Technician Hospital blood bank

#### Suggestions for improvement in management of SA cases

Suggestions for improvements were proposed at community, hospital and health systems levels. At the community level sensitization of the community to embrace malaria prevention strategies like use of insecticide treated nets, adoption of early health seeking behaviours, and donation of blood for hospital use. At hospital level regular in-service trainings to ensure that staff are familiar with clinical guidelines for managing SA; innovatively fill up staffing gaps with trainees and interns; and strengthen laboratory capacity to measure Hb. At health systems level there is need to strengthen blood transfusion services and the referral systems.

Quotes:

*‘… Communities should be sensitized about prevention strategies for SA. We should not wait to do firefighting.’* Hospital administrator

*‘…. they need to bring for us the machine for testing Hb and have Hb test done in outpatients department.’* Intern Doctor

*’.staff refresher course and training is needed to build their capacities to manage severe anemia better.’* Head nurse pediatric ward

## Discussion

In this study we found a disturbingly high rate of inappropriate use of blood transfusion in the management of anemic children in two referral hospitals in Uganda. More than half (55.5%) of the blood transfusions were given either presumptively (without documented pre-transfusion Hb done) or when a transfusion is not indicated (Hb > 6.0 g/dl). The major issues related to management of SA included unreliable availability of laboratory equipment and reagents to evaluate haemoglobin level and late presentation of SA children to hospital.

Our findings are similar to those of a Kenyan study that retrospectively reviewed transfusion requests from the paediatric ward of a tertiary hospital to the blood bank [12]. The authors found that 52% of the blood transfusions were requested based on clinical judgement without laboratory confirmation of the anemia. This high rate of inappropriate use of blood transfusion in resource limited setting is unacceptable because it leads to wastage of a scarce resource. In addition, giving blood inappropriately not only exposes children to unnecessary risks associated with blood transfusion like transfusion reactions etc. [18] but also deprives patients who are truly anemic opportunity to get life-saving blood transfusion [14].

In the study hospitals, a diagnosis of ‘severe anemia’ was the indication for blood transfusion in almost all (95%) children that were transfused. Conversely, children not diagnosed with SA were unlikely to be transfused, similar to a Tanzanian study where patients not identified by physicians in the emergency room to have severe anemia were not transfused [14]. Thus correct diagnosis of severe anemia is a critical step in the appropriate use of blood transfusion in resource limited settings.

The clinical diagnosis of SA is based on the finding of severe pallor on examination [19]. Compared to laboratory testing, diagnosis based on severe pallor overestimates the burden of SA due to its low specificity in detecting severe anemia [20, 21]. In this study a quarter of the children (25.3%) admitted in the two hospitals were assigned a diagnosis of severe anemia. However amongst SA children with Hb testing done, 26% had Hb > 6.0 g/dL suggesting that about a quarter of the children with clinical diagnosis of SA were not severely anemic and should not have been given the SA diagnosis.

One of the main reasons for the reliance on clinical diagnosis of SA was the lack of reliable equipment to measure Hb whenever needed. Overall Hb was measured in only 60% of suspected SA patients. Confirmation of SA diagnosis by Hb measurement should be available to the clinician as soon as possible to guide clinical decision on whether or not to transfuse. The standard method of measuring level of Hb involves using automated hematology analysers like the coulter counter machine [22]. However, hematology analysers are expensive, requiring ongoing costs for blood tubes and reagents, which is challenging in resource limited settings. They also require greater volumes of blood and a longer turnaround time which is not ideal in emergency conditions like SA where a rapid Hb measurement is required to make immediate management decisions. In this study the Coulter Counter machines in both hospitals frequently broke down because of overload or when they were operational they often lacked reagents. Hence Hbs were often not measured. The alternative would be to use point-of-care haemoglobinometers such as the HemoCue [22, 23] for estimating haemoglobin level. These devices require only a small finger prick for capillary blood sample and provide an estimation of Hb levels in real-time to facilitate objective clinical decision making by front line health workers. Moreover they can easily be used by front line health workers [24] and are fairly accurate if properly used [25]. Although point-of-care hemoglobin tests still require costly consumables like cuvettes, an investment in these point of care devices for measuring Hb would lead to improved inpatient care and help manage limited blood stocks in resource limited settings where mortality from severe anaemia remains unacceptably high.

Poor clinical habits contributed to inappropriate blood transfusions and lack of adherence to transfusion guidelines. In this study laboratory results were disregarded and at least 16% of the patients were transfused inappropriately with pre-transfusion Hb > 6 g/dl. This was lower than the 46% reported in children transfused with Hb > 6 g/dl in the Kenyan study [12]. The main reason given by the clinicians for none-adherence to blood transfusion guidelines was that some of the SA children present late and in critical condition so they prioritised giving blood transfusion without waiting for Hb testing or Hb result. This suggests that children transfused inappropriately were sicker than those deemed stable enough to wait for Hb testing. This is partly supported by the significant difference in inpatient mortality rate between SA children transfused as per guidelines compared to those transfused inappropriately on clinical grounds (*p* = 0.03). However many of the SA children reported to have come in critical conditions presented during the night shift when the staffing levels are low and composed mostly of junior level cadres. It is therefore more likely that the higher inpatient mortality in the SA children transfused on clinical grounds was because these children were misdiagnosed and inappropriately managed.

Efforts to improve transfusion practices should target clinicians who make the decisions of whether or not to transfuse. Transfusion guidelines also need to be tailored to take into consideration local needs and perceptions amongst health workers and the role of point of care bedside devices for hemoglobin measurements need to be explored. One such training modality is the emergency triage assessment and treatment plus admission care (ETAT+), which has been shown to result in improvement in knowledge [26], clinical skills [27], adherence to clinical guidelines [28] and clinical care [29]. To be effective, it is important for the training to be coupled with interventions that address gaps in health systems such as accessibility of care [30], supply chain issues, staffing levels, [31] remuneration and other such issues that affect staff morale and performance [32].

Delays in getting blood has been reported as one of the issues affecting clinical use of blood in other studies was not an issue in this study [33]. In this study, when blood was available, most of the children were able to receive the blood shortly upon request. This was probably due to the location of the blood banks within or close to the children’s wards. However, as has been the case in settings elsewhere [34, 35], blood stock-out was frequently reported despite the fact that both hospitals were blood collection centres. During blood stock-outs, severely anemic patients were advised to go to other health units where blood might be available. It is ironical that despite this chronic shortage, blood when available was given indiscriminately with disregard to transfusion guidelines. This tendency to transfuse on clinical grounds inflates the demand for blood leading to unnecessary blood transfusions and compounds the shortage of blood. In Uganda, 60% of an estimated 220,000 unit of blood collected annually is consumed by children [36]. If about a half is given inappropriately, as seen in this study, this translates to about 66,000 units of blood per year, more than the projected annual shortfall of 30,000 units annually [36].

Efforts to improve availability of blood for clinical use should include measures to reduce the demand for blood by addressing the causes of severe anemia. In our setting malaria was the most common etiological factor associated with SA. Efforts to control malaria should be strengthened as reduction in malaria rates has been reported to result in corresponding reduction of demand for blood [37].

There were several limitations in this study. Since this was a retrospective study of routine clinical records, it is possible that some of the Hb tests and results were not documented in the inpatient records. Our findings, therefore may underestimate the prevalence of laboratory confirmed SA. Secondly during stock out of blood suspected SA patients are often advised to go to other health units that might have blood. Such missed cases were not recorded so our findings only describe the transfusion practices when blood was available at the health facility. Thirdly, the care was not uniform and depended on the motivation, clinical skills and documentation of the clinician that attended to the patient. Nonetheless, by covering a large number of records over a 12 month period, we believe the data captured in this study presents the average care provided to SA children in Uganda. The major strength of the study is that it provides much needed documentation on blood use during routine clinical practice in resource limited settings.

## Conclusions

In conclusion, our study highlights the unacceptably high rate of inappropriate use of blood transfusion in the management of anemic children in Lira and Jinja hospitals in Uganda. Verification of Hb level by laboratory testing and training of health workers to adhere to transfusion guidelines could result in a substantial decrease in inappropriate blood transfusion, which is critical with the chronically low supply of blood in Ugandan hospitals. Future studies should explore the effect of adherence to clinical guidelines on inpatient mortality.

## Abbreviations

ETAT+: Emergency triage assessment treatment plus admission care
FDG: Focus group discussion
Hb: Hemoglobin
HIV: Human immunodeficiency virus
ICD: International classifications of diseases
KII: Key informant interview
SA: Severe anemia
